# Tight Junction Component Occludin Binds to FIP5 to Regulate Endosome Trafficking and Mitotic Spindle Function

**DOI:** 10.1002/advs.202308822

**Published:** 2024-06-17

**Authors:** Zichao Zhang, Jing Chen, Rongze Ma, Chongshen Xu, Yunzhe Lu, Jiecan Zhou, Kun Xia, Pengfei Lu

**Affiliations:** ^1^ MOE Key Lab of Rare Pediatric Diseases Hengyang Medical School University of South China Hengyang China; ^2^ Institute of Cytology and Genetics School of Basic Medical Sciences Hengyang Medical School University of South China Hengyang China; ^3^ Institute for Future Sciences Hengyang Medical School University of South China Changsha China; ^4^ School of Life Science and Technology ShanghaiTech University Shanghai China; ^5^ The First Affiliated Hospital Hengyang Medical School University of South China Hengyang China

**Keywords:** branching morphogenesis, centrosome, epithelial migration, RAB proteins, spindle organization, vertebrate‐specific genes, vesicular trafficking

## Abstract

The genetic basis of vertebrate emergence during metazoan evolution has remained largely unknown. Understanding vertebrate‐specific genes, such as the tight junction protein Occludin (*Ocln*), may help answer this question. Here, it is shown that mammary glands lacking *Ocln* exhibit retarded epithelial branching, owing to reduced cell proliferation and surface expansion. Interestingly, *Ocln* regulates mitotic spindle orientation and function, and its loss leads to a range of defects, including prolonged prophase and failed nuclear and/or cytoplasmic division. Mechanistically, *Ocln* binds to the RabGTPase‐11 adaptor FIP5 and recruits recycling endosomes to the centrosome to participate in spindle assembly and function. FIP5 loss recapitulates *Ocln* null, leading to prolonged prophase, reduced cell proliferation, and retarded epithelial branching. These results identify a novel role in OCLN‐mediated endosomal trafficking and potentially highlight its involvement in mediating membranous vesicle trafficking and function, which is evolutionarily conserved and essential.

## Graphical abstract

Mammary glands lacking *Occludin* exhibit retarded epithelial branching, owing to reduced cell proliferation. Interestingly, *Occludin* regulates mitotic spindle orientation and function, and its loss leads to a range of defects, including prolonged prophase and failed nuclear and/or cytoplasmic division. Mechanistically, *Ocln* binds to FIP5 and recruits recycling endosomes to the centrosome to participate in spindle assembly and function.

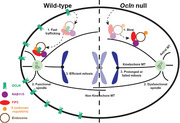

## 1 Introduction

Despite being a major event during metazoan evolution, the genetic basis of vertebrate emergence has remained poorly understood.^[^ ^1^, ^2^ ^]^ One way to better understand the process is to focus on vertebrate‐specific genes (VSGs), accounting for ≈20% of the vertebrate genome.^[^ ^3^ ^]^ Of the VSGs, the tight junctions (TJ) component Occludin (*Ocln*) could be an ideal candidate to study because it is essential for the physiological function of a multitude of vertebrate organs.^[^ ^4^, ^5^ ^]^ Moreover, as the only member of its family,^[^ ^6^ ^]^ it is free from redundancy issues often associated with multi‐member gene families, thus making its functional determination more readily, especially regarding its most fundamental cell biological functions that were essential for vertebrate evolution.

Although the initial evidence indicated that *Ocln*, as its name suggests, is essential for TJ's barrier functions, mouse embryonic stem cells lacking *Ocln* are capable of forming functional TJs, and mutant embryos develop normally to birth.^[^ ^7^, ^8^ ^]^ However, mutant mice and patients lacking *Ocln* function exhibit various organ deficiencies, including deafness, male infertility, lactation failure, microencephaly, brain calcification, and mental abnormalities.^[^ ^8^ ^]^ These abnormalities provide us with an excellent opportunity to understand not only the molecular etiologies underlying the illnesses, but also *Ocln* functions in different developmental contexts.

Recent studies have shown that *Ocln* promotes cortical brain development during embryogenesis, and its absence leads to microencephaly in *Ocln*‐null mice.^[^ ^9^ ^]^ We previously demonstrated that OCLN binds to the SNARE complex to facilitate milk protein secretion^[^ ^10^ ^]^ and regulates lipid secretion, an independent process from protein secretion,^[^ ^11^ ^]^ thus explaining why mutant female mice experience lactation failure and are unable to nurse their young. However, it has become apparent that a comprehensive analysis of *Ocln* function in diverse biological contexts is necessary before a common theme of *Ocln*’s conserved subcellular function is available.

During the course of our investigations, we observed that epithelial branching is slower than normal in *Ocln* mutant mice, providing a promising opportunity to potentially identify a novel function of OCLN. In this current study, we present a characterization of the epithelial branching defect observed in the mutant mice and delve into the essential cell biological function of *Ocln* responsible for this defect.

## 2 Results

### 2.1 *Ocln* Promotes Epithelial Expansion During Mammary Gland Branching Morphogenesis

**Figure 1:**
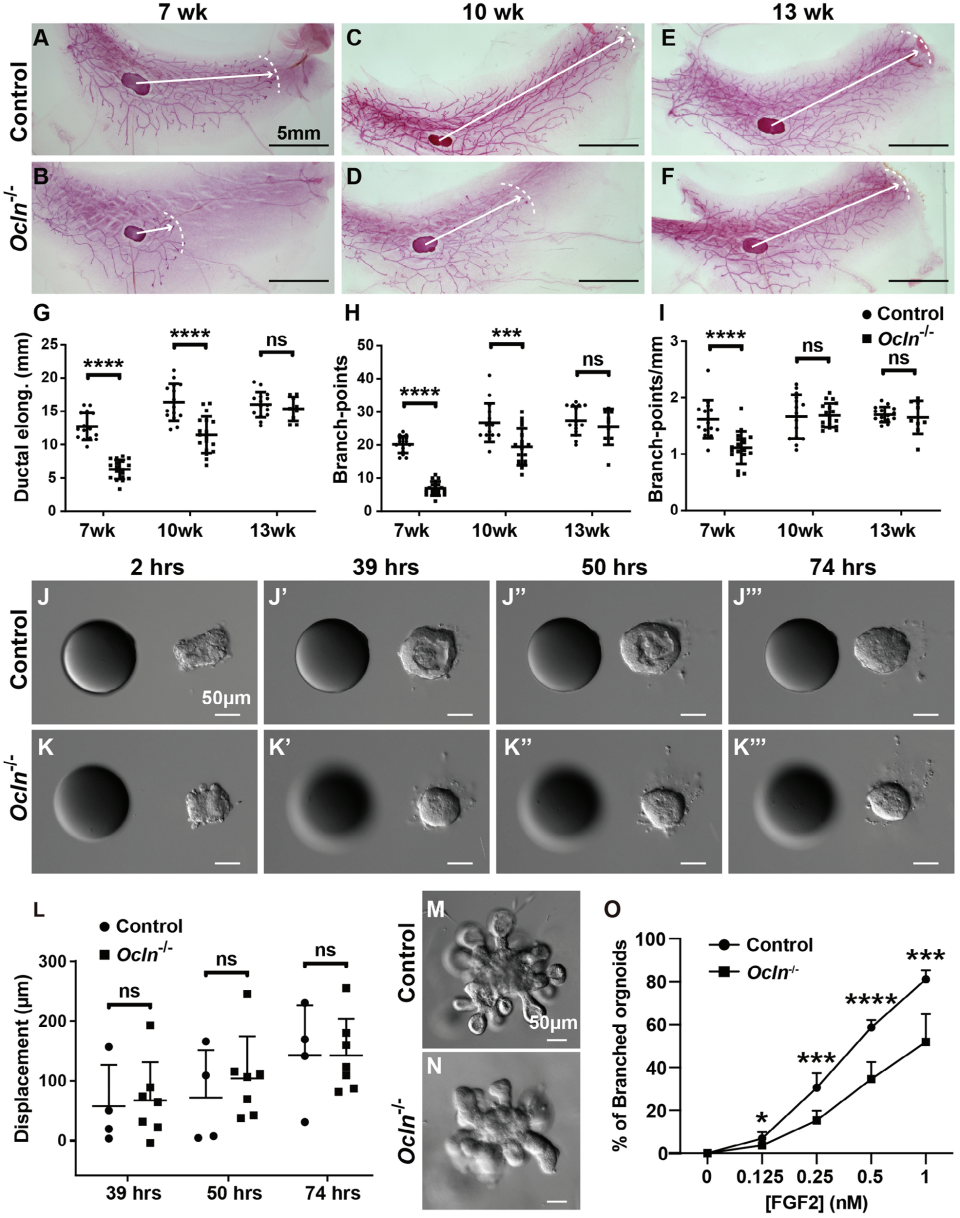
*Ocln* promotes epithelial expansion during mammary gland branching morphogenesis. A–F) Branching trees of the #4 mammary glands, as revealed by Carmine Red staining of whole‐mount mammary glands, at the 7‐week (A,B), 10‐week (C,D), and 13‐week stages (E,F). (A,C,E) Glands from control (*Ocln* ^−/+^) mice; (B,D,F) glands from *Ocln* null (*Ocln* ^−/−^) mice. Arrows indicate the extent of ductal penetration in the fat pad. The dotted white line illustrates the epithelial invasion front. G–I) Quantitative comparisons of ductal elongation, branch‐points, and branching points per millimeter between control and mutant glands. Plots show mean ± SD. Scale bars: 5 mm. The number of female mice used was as follows: control (*n* = 15), and *Ocln* ^−/−^ (*n* ≥ 17) at the 7‐week stage; control (*n* ≥ 14), and *Ocln* ^−/−^ (*n* = 18) at the 10‐wk stage; control (*n* = 15), and *Ocln* ^−/−^ (*n* = 9) at the 13‐wk stage. J‐K**’’’**) Time course of control (J‐J’’’) and *Ocln* null (K‐K’’’) organoid migration toward beads pre‐soaked in FGF10. Heparin acrylic beads of ≈100 µm in diameter were juxtaposed with organoids at a distance of ≈100 µm. Number of organoids analyzed (control, *n* = 4; *Ocln* ^−/−^, *n* = 7). A *t*‐test was used. Scale bars: 50 µm. L) Quantification of the total displacement of control and *Ocln* null organoids. Data are mean ± SD. Statistical analysis was performed using an unpaired student *t*‐test. M–O) in vitro branching assay in which control (M) and *Ocln* null (N) organoids were subjected to cultures in basal medium containing FGF2. When stimulated by FGF2 at progressively higher concentrations from 0.125 to 1 nm, a progressively higher percentage of organoids underwent branching. Scale bars: 50 µm. (O) Quantitative comparisons of control and *Ocln* null organoids in their ability to undergo epithelial branching in vitro. At least 100–150 organoids were examined for each treatment condition. ns = not significant; ^*^ *p* < 0.05; ^**^ *p* < 0.01; ^***^ *p* < 0.001; ^****^ *p* < 0.0001.

Using Carmine‐stained whole‐mount mammary glands, we assessed the impact of *Ocln* loss on epithelial development at different stages, including 7 and 10 weeks (active branching period) and 13 weeks (branching completion) (**Figure** **1**A–F). In control mammary glands, there was an increase in ductal elongation and penetration into the fat pad from 7 to 13 weeks (Figure 1A,C,E). Moreover, the number of branches and branch points progressively increased with age, with the three longest epithelial branches averaging 28 branch points at 13 weeks (Figure 1G,H). Despite the increase in ductal elongation and the total number of branch points, the branch points per millimeter remained relatively stable at ≈1.8 branch points mm^−1^ (Figure 1I).

Compared to the control glands, both ductal elongation and the total number of branch points in *Ocln* null mammary glands were reduced (Figure 1B–F,G,H). Specifically, ductal elongation in *Ocln* null glands was ≈50% and 25% lower at 7 and 10 weeks, respectively, than in control glands. No difference in ductal elongation was observed at 13 weeks, likely due to branching completion in both control and *Ocln* null glands (Figure 1E,F). The number of branch points in null glands was also ≈75% and 25% lower than in control glands at 7 and 10 weeks, respectively, but similar to those in control glands at 13 weeks (Figure 1H). The branch points per unit in null glands were fewer than in control glands at 7 weeks but similar to control glands at both 10 and 13 weeks (Figure 1I).

One possible explanation for the observed phenotype is that *Ocln* null glands suffer from defective epithelial migration, an essential aspect of branching morphogenesis.^[^ ^12^ ^]^ To test this possibility, we subjected control and *Ocln* null mammary epithelia to a 3D in vitro migration assay that we recently demonstrated to recapitulate the in vivo process.^[^ ^12^, ^13^ ^]^ Thus, mammary organoids from control and *Ocln* null glands were placed near a heparin sulfate bead pre‐soaked in FGF10, the most highly expressed FGF ligand in the mammary stroma,^[^ ^13^, ^14^, ^15^ ^]^ and aggregate migration was analyzed over the next three days. Interestingly, we found that *Ocln* null organoids showed similar migration abilities to their control counterparts during the analysis period (Figure 1J–L), suggesting that defective migration is unlikely the cause of retarded branching in *Ocln* null glands.

Alternatively, *Ocln* null epithelium may suffer from reduced surface expansion or other migration‐independent aspects of branching morphogenesis, which could be assessed by an FGF2‐based branching assay.^[^ ^13^, ^16^ ^]^ As expected, we found that control mammary epithelial cells (MECs) formed branched structures at progressively higher percentages as the concentration of FGF2 increased (Figure 1M,O). Interestingly, we found that the branching kinetics of *Ocln* null epithelium were lower than the control epithelium (Figure 1N,O). These in vitro data collectively demonstrate that *Ocln* mutant epithelium exhibits defective epithelial expansion.

It is possible that mutant epithelium may have a compromised response to FGF signaling due to *Ocln* loss, resulting in a failure to undergo FGF2‐based epithelial expansion. Therefore, we measured the mRNA expression of several FGF signaling target genes, including *Etv4*, *Etv5*, and *Mkp3*,^[^ ^14^, ^15^ ^]^ in mammary gland epithelium with or without *Ocln* function. We found that mutant MECs did not show significant differences in mRNA expression of *Etv4*, *Etv5*, and *Mkp3* compared to control MECs (Figure S1A–C, Supporting Information). These data indicate that MECs lacking *Ocln* function are not desensitized to FGF signaling activities.

Taken together, the above data demonstrate that epithelial branching is delayed in *Ocln* null glands as a result of *Ocln* function in promoting epithelial expansion rather than collective migration.

### 2.2 *Ocln* Regulates Mitosis of Luminal Epithelial Cells

**Figure 2:**
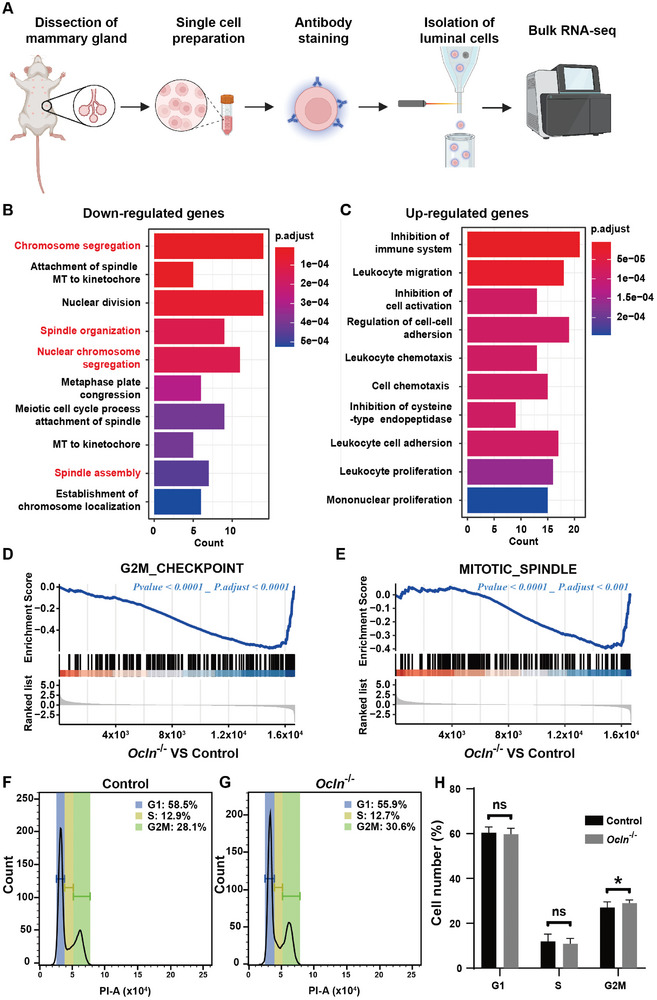
*Ocln* regulates the mitosis of luminal epithelial cells. A) Diagram illustrating the experimental procedure for bulk RNA sequencing of control and *Ocln* ^−/−^ mammary glands to determine transcriptional changes in luminal epithelial cells. B,C) Gene Ontology (GO) analysis of the main downregulated (B) or upregulated (C) pathways in *Ocln* null luminal epithelial cells of the mammary gland. D,E) Gene Set Enrichment Analysis (GSEA) of pathways related to the G2M checkpoint (D) and the mitotic spindle (E) in *Ocln* null mammary glands compared to control mammary glands. Rank lists denote fold‐change values after data processing. F–H) Cell cycle analysis of control (F) and *Ocln* null (G) luminal epithelial cells based on flow cytometry. (H) Quantification of the percentages of cells in different phases of the cell cycle (both control and *Ocln* ^−/−^, *n* = 10). Note: “ns” indicates not significant; ^*^ *p* < 0.05.

To determine the underlying causes of reduced epithelial expansion resulting from *Ocln* loss, we utilized bulk RNA sequencing (RNA‐seq) to analyze changes in gene expression at the transcriptomic level in *Ocln* null cells compared to normal cells. For this purpose, we used FACS to sort luminal cells from control and *Ocln* null mammary glands at the 7‐week stage, when the branching phenotype was readily observed (Figure 1), and then prepared cDNA libraries and subjected them to sequencing (**Figure** **2**A).

Initially, our focus was on transcriptomic changes in the luminal cells caused by *Ocln* loss. Gene ontology (GO) analyses revealed significant enrichment of down‐regulated pathways, particularly those involved in mitotic processes such as chromosome segregation, spindle organization, and function (Figure 2B). However, no clear trend was observed in the upregulated pathways (Figure 2C). Consistent with these findings, Gene Set Enrichment Analysis (GSEA) demonstrated reduced expression of genes in pathways regulating the G2M checkpoint and mitotic spindle (Figure 2D,E). These data indicate that *Ocln* loss leads to reduced proliferation in luminal cells, which aligns with the observed branching phenotype.

During normal development, the ratio of basal and luminal cells remains relatively constant.^[^ ^17^ ^]^ Thus, we hypothesized that the reduced luminal cell proliferation would impact this ratio. To test this prediction, we employed FACS to analyze the percentages of basal and luminal cells in the overall preparation of control and *Ocln* null mammary glands. Consistent with our hypothesis, we observed a decrease in the luminal cell percentage, accompanied by an increase in the basal cell percentage (Figure S2A,B, Supporting Information). Consequently, the luminal‐to‐basal ratio in the *Ocln* null glands was reduced from 3 to 2 compared to the control glands (Figure S2C, Supporting Information).

Subsequently, we utilized flow cytometry to investigate cell cycle defects in *Ocln* null luminal cells. To achieve this, we stained the control and *Ocln* null luminal cells with the nuclear dye propidium iodide (PI) and analyzed the samples using a flow cytometer. Our findings revealed that in normal glands, luminal cells in the G2M phase accounted for ≈28.1% (Figure 2F), whereas they accounted for 30.6% of the total cell population of the *Ocln* null glands. No significant changes were observed in the percentages of cells in other phases of the cell cycle, including the G1 and S phases (Figure 2H).

Notably, despite a relatively modest change in the percentage of cells in mitosis, *Ocln* null glands exhibited pronounced epithelial branching phenotypes, illustrating the sensitivity and effectiveness of utilizing branching as a quantifiable measure of defects during epithelial morphogenesis of the mammary gland. Moreover, it is possible that our assay, which examined cells of the entire epithelium, most of which are in the duct, rather than in the TEBs where cells are proliferating, underestimated the effect that OCLN loss has on proliferating cells. Together, these results suggest that a significant proportion of *Ocln* null cells are arrested in the G2M phase, leading to reduced proliferation of luminal cells.

### 2.3 *Ocln* Regulates Mitotic Spindle Organization and Function

**Figure 3:**
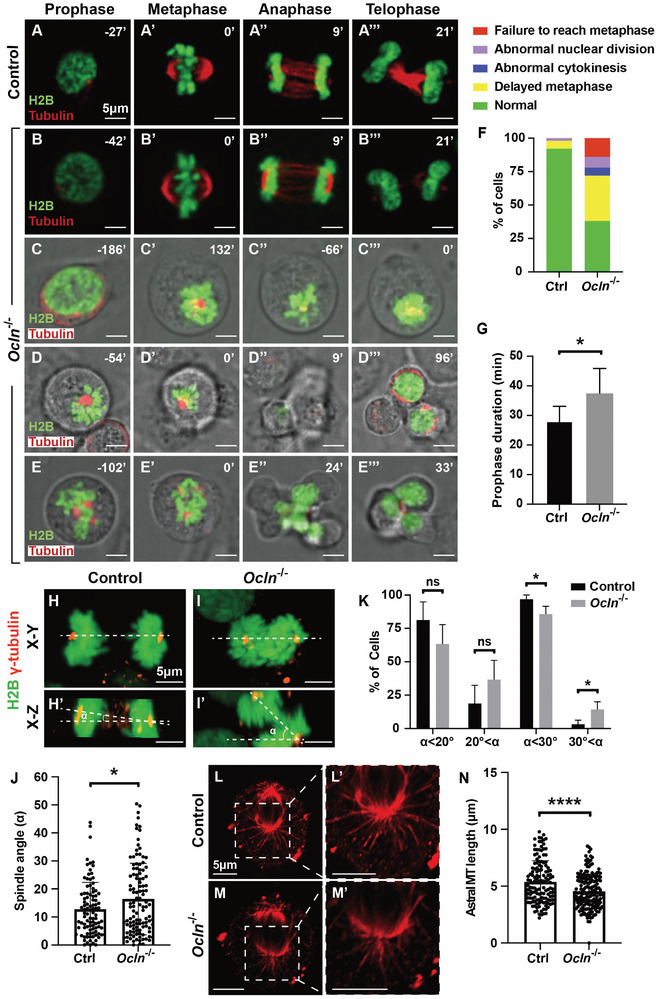
*Ocln* regulates mitotic spindle organization and function. A–G) Time‐lapse video recordings of mitotic progression in control cells (A‐A’’’) and *Ocln* null HC11 cells (B‐E’’’). Specific time points were retrospectively assigned following each experiment. Time zero (0′) was set at the middle of metaphase in control (A’) and *Ocln* null cells (B’), while it was arbitrary for *Ocln* null cells that failed to complete mitosis (C‐E’’’). (F) Quantification of different mitosis categories in control and *Ocln* null cells. (G) Quantification of the duration between prophase onset and metaphase in control and *Ocln* null cells. The number of cells analyzed: control (*n* = 51); *Ocln* ^−/−^ (*n* = 50). A *t*‐test was used; ^*^ *p* < 0.05. Scale bars: 5 µm. H–K) The mitotic spindle angle was determined by measuring the angle between centrosomes (marked by γ‐tubulin immunofluorescence in red) in the horizontal X‐Y plane (H,I) and vertical X‐Z plane (H’,I’). (J) Quantification of spindle angles; (K) angle distributions in control (*n* = 96) and *Ocln^−/−^* (*n* = 114) cells. A *t*‐test was used; ns, not significant; ^*^ *p* < 0.05. L–N) Lengths of astral microtubules (α‐tubulin immunofluorescence in red) measured in control (L,L’) and *Ocln* null (M,M’) cells and quantified (N). Number of cells used: control (*n* = 140); *Ocln* ^−/−^ (*n* = 181). Scale bars: 5 µm. A *t*‐test was used; ^****^ *p* < 0.0001.

To determine the detailed mitotic defects due to *Ocln* loss, we generated an HC11 clone of cells lacking *Ocln* function based on the CRISPR‐Cas9 technique (Figure S3A,B, Supporting Information). Next, we utilized time‐lapse live imaging and tracked the dynamics of the mitotic spindle and chromosomes across various stages of mitotic progression. Our findings revealed that the majority (90%) of luminal cells in the control gland completed mitosis in ≈48 min, with only ≈5.9% experiencing prolonged mitosis, and a negligible percentage exhibiting abnormal nuclear division (**Figure** **3**A‐A′″F). In contrast, only ≈38% of *Ocln* null luminal cells displayed normal mitotic progression, while the remaining, ≈62% exhibited varying degrees of mitotic defects. Specifically, ≈34% of mutant luminal cells had prolonged mitosis, while ≈28% suffered from more severe defects, including ≈14% being unable to reach metaphase, and the rest exhibiting abnormal nuclear and/or cytoplasmic divisions (Figure 3B–F; Movies [Link], [Link], [Link], [Link], Supporting Information). Compared to control luminal cells, whose prophase lasted ≈27 min, *Ocln* null cells with prolonged mitosis had on average a prophase of ≈37 min, representing a 37% increase (Figure 3G).

Together, these results indicate that *Ocln* regulates mitotic progression, including from prophase to metaphase. In its absence, mutant cells experience prolonged prophase or, to a lesser extent, fail to reach metaphase or effectively divide the chromosomes. To further assess the role of *Ocln* in regulating the mitotic spindle, we stained the centrosomes and chromosomes and measured the spindle angle. We observed that in control cells, the majority (≈97%) of cells displayed an angle of ˂30 degrees, whereas this percentage was reduced to ≈86% in *Ocln* null cells, which also showed a wider spindle angle than control cells (Figure 3H–K). Furthermore, using an alpha‐tubulin antibody, we quantified the lengths of astral microtubules and found that they were significantly shorter in *Ocln* null cells compared to control cells (Figure 3L–N).

Taken together, these findings demonstrate the essential role of *Ocln* in mitotic spindle assembly and function. In its absence, the mitotic spindle becomes abnormal and fails to function properly, resulting in delayed or complete failure to reach metaphase or effectively separate the chromosomes.

### 2.4 *Ocln* Is a Resident Component on Mitotic Endosomes

**Figure 4:**
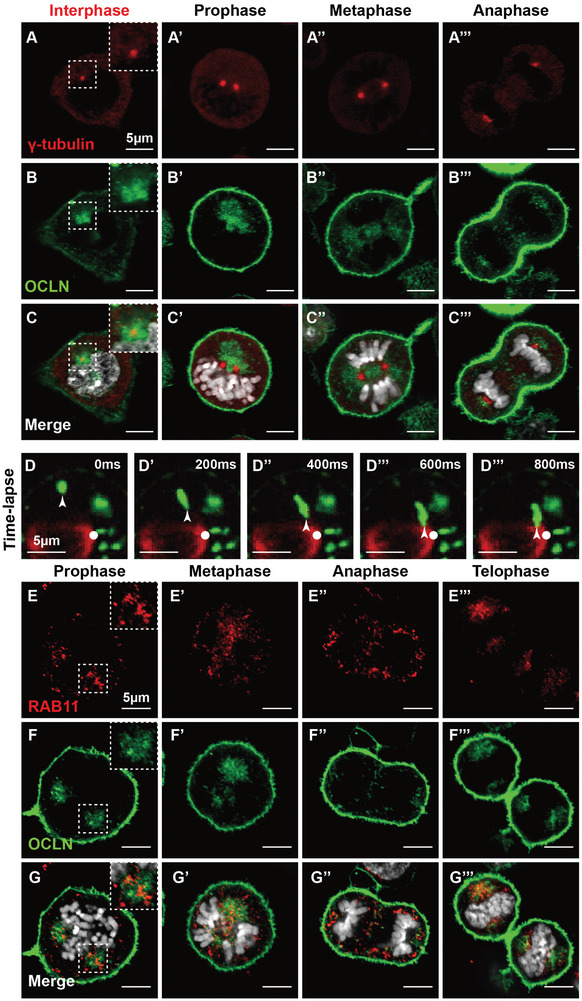
OCLN is a component of mitotic endosomes. A–C’’’) Time‐course of OCLN expression indicated by eGFP‐OCLN (green), during different phases of the cell cycle in HC11 cells. Centrosomes and chromosomes are marked by γ‐tubulin (red) and DAPI immunofluorescence, respectively. Scale bars: 5 µm. D‐D’’’) Tracking of BFP‐OCLN protein (green) trafficking to the centrosome through time‐lapse video recording. The arrowhead indicates an OCLN‐positive vesicle being tracked during the recording, while the centrosome is denoted by a white dot at its expected position—the bottom of the mitotic spindle, marked by α‐tubulin immunofluorescence (red). Scale bars: 5 µm. E‐H’’’) Co‐localization analysis of RAB11 (red, F‐F’’’) and eGFP‐OCLN (green, G‐G’’’) through time‐course fluorescent confocal imaging during mitosis. Chromosomes (DAPI, white) were superimposed on the RAB5 and OCLN signals in the merged images (H–H’’’). Scale bars: 5 µm.

To determine how OCLN, a transmembrane protein expressed on tight junctions and the plasma membrane, regulates mitotic spindle organization, we investigated its localization during mitosis. For this purpose, we generated a fusion protein with eGFP fused in‐frame with OCLN at the N‐terminus. Lentivirus carrying the expression construct was used to infect the mammary epithelial cell line HC11 cells. We recorded eGFP‐OCLN fluorescence, as well as immunofluorescence for γ‐tubulin and DAPI nuclear fluorescent dye, which respectively mark the centrosome and chromosomes, at different phases of the cell cycle. Our findings indicate that, apart from its presence in the plasma membrane, OCLN is also expressed around the centrosome throughout the cell cycle stages (**Figure** **4**A–C′″). Notably, its presence around the centrosome was particularly evident during prophase, and the fluorescence intensity gradually decreased from metaphase onward (compare Figure 4B′ with B″ and B′″).

To investigate how OCLN congregates around the centrosome during prophase, we conducted a time‐lapse video recording of BFP‐OCLN, where OCLN protein was tagged by the blue fluorescent protein (BFP) at the N‐terminus, in HC11 cells stained with a live microtubule dye. We observed that OCLN‐positive aggregates, likely representing membranous vesicles with a diameter of ≈1 µm, migrated toward the centrosome during prophase (Figure 4D‐D’’’; Movies S5,S6). Based on their sizes, we speculate that these vesicles are endosomes.

To verify this possibility, we initially examined the co‐localization of transferrin receptor (TfR), which is also a membrane protein, with OCLN. As expected, TfR was present in the plasma membrane, but due to its constant recycling, it was also prominently present in numerous sparsely distributed vesicles, i.e., recycling endosomes as previously reported,^[^ ^18^ ^]^ throughout the mitotic stages (Figure S4A‐A’’’, Supporting Information). Interestingly, we observed a substantial overlap between TfR‐positive and OCLN‐positive vesicles (Figure S4A–C’’’, Supporting Information). Next, we investigated the relationship between OCLN‐positive vesicles and RAB5‐positive vesicles, which are early endosomes known to play a role in spindle orientation and chromosome segregation.^[^ ^19^, ^20^ ^]^ We observed a broad overlap between RAB5, and OCLN‐positive vesicles compared to that between TfR and OCLN (Figure S4E,F’’’, Supporting Information).

Finally, we examined the association between OCLN‐positive vesicles and RAB7‐positive vesicles, which are late endosomes, as well as RAB11‐positive vesicles, which are associated with a group of recycling endosomes that, similar to RAB5, are involved in regulating spindle organization and function.^[^ ^21^ ^]^ In both cases, we detected varying degrees of overlap between RAB7‐ (Figure S4G–I’’’, Supporting Information) or RAB11‐positive endosomes and OCLN‐positive endosomes (Figure 4E–G’’’).

In summary, our findings show that OCLN exhibits varying degrees of overlap with endosomes, including those containing RAB5, RAB7, and RAB11, some of which are known to regulate mitotic progression. Together, these data indicate that OCLN resides on mitotic endosomes essential for cell cycle progression.

### 2.5 *Ocln* Binds to the Vesicular Trafficking Regulator *Fip5*

**Figure 5:**
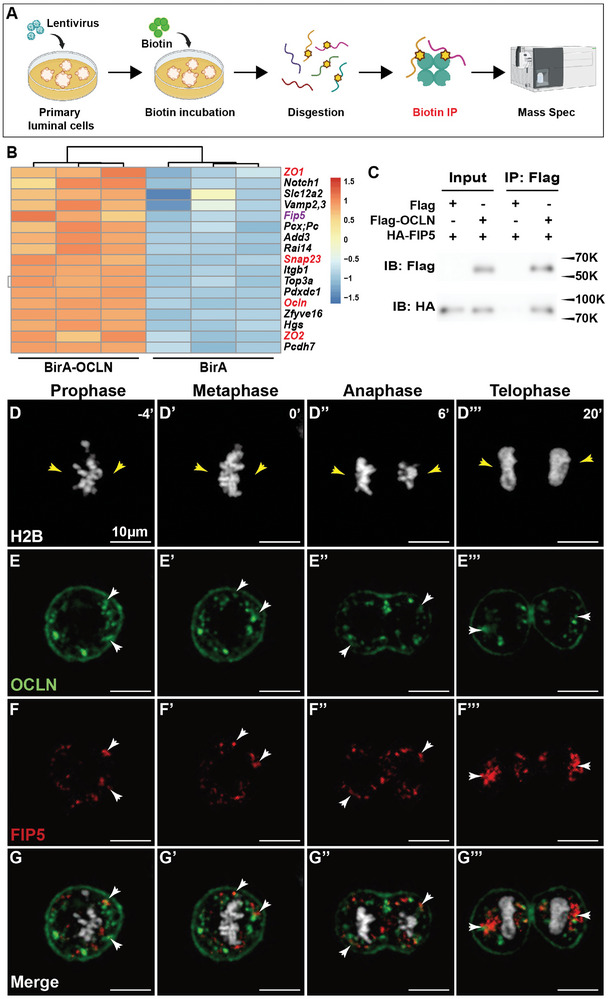
OCLN binds to the vesicular trafficking regulator FIP5. A) Schematic diagram illustrating the design and workflow of the BioID assay. Expression plasmids carrying the Biotinylase (BirA)‐OCLN fusion protein or BirA alone were used to transfect primary mammary epithelial luminal cells for the purpose of screening OCLN protein partners. B) Top candidate protein partners of OCLN identified through the BioID screen. Notably, the tight junction constituents of ZO1, ZO2, the established binding partner SNAP23, and OCLN itself were all present in the list, thus validating the efficacy of the screen. C) Detection of protein interaction between OCLN and FIP5 using co‐IP assays. OCLN was tagged with Flag protein, while FIP5 was tagged with HA. Immunoprecipitation was performed using an anti‐Flag antibody, followed by Western blot analysis with an anti‐HA antibody. Molecular weight markers (in kilodaltons) are indicated by numbers. D–G’’’) Co‐localization analysis of OCLN (BFP‐OCLN, green, E‐E’’’) and FIP5 (mCherry‐FIP5, red, F‐F’’’) through time‐lapse imaging during mitosis. Chromosomes (white) were labeled with H2B‐eGFP (D‐D’’’). Yellow arrowheads indicate the anticipated positions of centrosomes, deduced from the shapes of condensed chromosomes during these mitotic stages. White arrowheads denote vesicles carrying both OCLN and FIP5 as revealed by the mitotic time‐lapse. Scale bars: 5 µm.

To determine the mechanism by which *Ocln* regulates mitotic spindle function, we utilized the proximity‐dependent biotin identification (BioID) labeling method^[^ ^22^ ^]^ to identify protein partners of OCLN that might play a role in this biological process. Therefore, we generated a construct containing the Biotinylase‐OCLN (BirA‐OCLN) fusion protein sequence, along with a control construct carrying BirA alone for internal comparison (**Figure** **5**A). These constructs were then transfected into primary luminal cells, and subsequent steps of the BioID experiments were conducted (Figure 5A).

Among the top hits from the BioID screen, we identified several known protein partners of OCLN, including the tight junction proteins ZO1 and ZO2, as well as Vamps and Snap23, known binding partners of the SNARE complex, which we have previously demonstrated^[^ ^10^ ^]^ (Figure 5B; Figure S5A,B, Supporting Information). The presence of these known OCLN partners validates the effectiveness of the BioID method in identifying novel protein partners of OCLN. However, we did not observe components of the centrosome, kinetochore, and spindle machinery, nor did we detect any RabGTPases, especially RAB5 and RAB11 that are known to regulate mitosis (Table S1, Supporting Information).

Among the candidate OCLN protein partners that were not previously reported, FIP5 (RAB11FIP5) stood out as a member of the RAB11FIP family^[^ ^18^, ^23^ ^]^ (Figure S5B,C, Supporting Information). Therefore, one possibility was that OCLN may regulate mitotic spindle organization and function by binding to FIP5, which interacts with RAB11 or other mitotic RABs.^[^ ^24^ ^]^ To test this hypothesis, we employed two independent methods to investigate the binding between OCLN and FIP5. In the first method, we tagged the N‐terminus of OCLN and FIP5 proteins with FLAG and HA peptides, respectively. Co‐immunoprecipitation (Co‐IP) was then performed using HEK 293T cells due to their high transfectability, and the results confirmed the binding of OCLN to FIP5 (Figure 5C) in this in vitro study.

To further examine if OCLN and FIP5 proteins co‐localize within living cells, we fused BFP to the N‐terminus of OCLN and mCherry to the N‐terminus of FIP5. Lentiviral constructs expressing these fusion proteins were introduced into HC11 cells expressing an H2B‐eGFP knock‐in construct, followed by fluorescence confocal microscopy analysis. We found a significant overlap between OCLN‐positive and FIP5‐positive vesicles during both prophase and the rest of the mitotic phases (Figure 5D–G’’’; Movie S7, Supporting Information). Taken together, our data demonstrate that OCLN binds to FIP5, a known partner of the mitotic RAB11.

### 2.6 *Fip5* Regulates Mitotic Spindle Function and Epithelial Branching in the Mammary Gland

**Figure 6:**
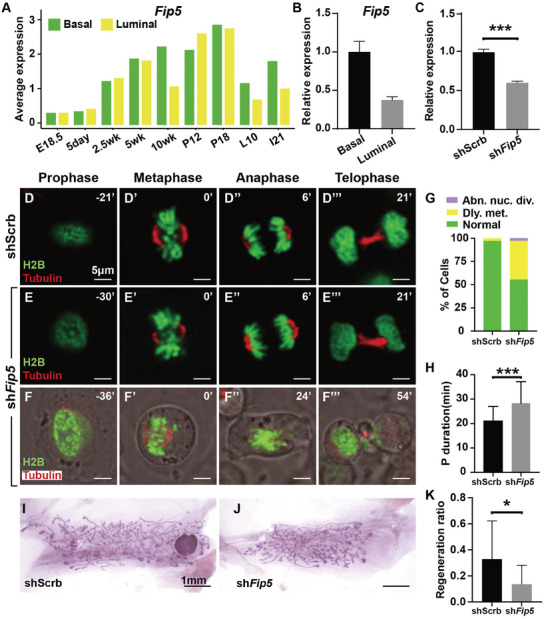
*Fip5* regulates mitotic spindle function and epithelial branching in the mammary gland. A) mRNA expression of *Fip5* in basal and luminal cells at different stages of mouse mammary gland development based on mining scRNA‐seq datasets. B) Relative expression of *Fip5* in sorted basal and luminal cells. C) Efficiency of *Fip5* knockdown via shRNA, as measured by its relative mRNA expression in HC11 cells. D‐H) Time‐lapse video recording of the mitotic progression in control, scrambled shRNA (shScrb) (D‐D’’’) and *Fip5* knockdown (sh*Fip5*) (E‐F’’’) luminal cells. Note that the specific time‐points indicated were set retrospectively at the conclusion of each experiment, with time zero (0′) being set at the middle of the metaphase of shScrb cells (D’) and sh*Fip5* cells (E‐F’’’). (G) Quantification of different categories, including abnormal nuclear division (Abn. nuc. div) and delayed metaphase (Dly. met.), of mitosis in shScrb and sh*Fip5* cells. (H) Quantification of the duration between prophase onset and metaphase in shScrb and sh*Fip5* cells. The number of cells analyzed (shScrb, *n* = 35; sh*Fip5*, *n* = 36). A *t*‐test was used; ^***^ *p* < 0.001. Scale bars: 5 µm. I–K) The whole mount of the epithelial network was revealed by Carmine staining of control (I) and *Fip5‐*knockdown mammary gland (J). (K) Quantification of the regeneration ratio of mammary glands. The number of glands analyzed (both shScrb and sh*Fip5*, *n* = 13). A *t*‐test was used; ^*^ *p* < 0.01. Scale bars: 1 mm.

Using the published scRNA‐seq databases,^[^ ^25^ ^]^ we found that *Fip5* is expressed in both basal and luminal cells during mammary gland development, particularly starting from around the onset of puberty at two‐and‐a‐half weeks of age when epithelial morphogenesis becomes active (**Figure** **6**A). We performed qPCR and confirmed the expression of *Fip5* in basal and luminal cells of the seven‐week‐old mammary gland (Figure 6B), which is characterized by active epithelial branching and expansion. By employing a lentiviral construct expressing *Fip5* shRNA (sh‐*Fip5*), we reduced its mRNA expression by ≈40% in HC11 cells. We were unable to obtain shRNAs that could more efficiently knock down *Fip5* mRNA expression, presumably because *Fip5* is essential for the proliferation and/or survival of HC11 cells.

To investigate the specific mitotic defects in *Fip5* knockdown HC11 cells, we conducted time‐lapse live imaging to track the dynamics of the mitotic spindle and chromosomes across various stages of mitotic progression. Control HC11 cells exhibited minimal delay in mitosis, with nearly all completing the process within 42 min (Figure 6D,G), which curiously is slightly shorter than the prophase duration shown by the control cells in the OCLN experiment (Figure 3A), presumably as a result of shRNA lentiviral infection. In contrast, only ≈56% of *Fip5* knockdown HC11 cells exhibited normal mitotic progression, while ≈42% experienced delayed mitosis of 51 min (compared to 63 min in *Ocln* null mentioned above), and ≈2% displayed abnormal nuclear division defects (Figure 6E–G; Movies S8 and S9, Supporting Information). Furthermore, *Fip5* knockdown cells demonstrated an extended prophase of ≈7 min, representing a 33% increase compared to shScrb (scrambled shRNA) control luminal cells, which typically lasted ≈21 min (Figure 6H). These results indicate that *Fip5*, similar to *Ocln*, plays a role in regulating mitotic progression, particularly from prophase to metaphase.

To examine the involvement of *Fip5* in the mitotic spindle, we conducted staining for centrosomes and chromosomes and measured the spindle angle. However, we did not observe a statistically significant change in the mitotic angle in *Fip5* knockdown cells (Supplementary Figure 6A,B).

To investigate the function of *Fip5* during epithelial branching, we harvested primary MECs and transfected them with either control shScrb or experimental sh*Fip5* constructs. These transfected cells were then transplanted into the cleared fat pad of nude mice. Our findings indicate that sh*Fip5*‐mediated knockdown resulted in a reduction of epithelial regeneration by ≈60% (Figure 6I–K). Subsequently, using flow cytometry, we analyzed the cell cycle of control and *Fip5* knockdown luminal cells. For this purpose, we stained the control and *Fip5* knockdown cells with PI and processed the sample through the flow machine. The results revealed that in normal glands, luminal cells in the G2M phase accounted for ≈23.9% (Supplementary Figure 6C–E). However, in the *Fip5* knockdown glands, these cells accounted for 27.6% of the total cell population, representing a 3.7% increase. This increase led to slight but significant alterations in the percentages of cells in other phases of the cell cycle, including the G1 and S phases (Figure S6C–E, Supporting Information).

To determine whether *Fip5* shRNA may have any off‐target effects, we performed a “rescue” experiment in which *Fip5* was forcibly expressed in sh‐*Fip5* cells. We then measured how this affected *Fip5* mRNA expression and the cell cycle. We found that forceful re‐expression of *Fip5* greatly upregulated *Fip5* mRNA expression, and the cell cycle defects observed in sh‐*Fip5* cells were restored to normal (Figure S6F,G, Supporting Information). The results thus suggest that there are no obvious off‐target effects caused by the sh‐*Fip*5 expression.

Taken together, our data demonstrate that *Fip5* plays a role in regulating mitotic spindle function and epithelial branching in the mammary gland.

### 2.7 *Ocln* Promotes Endosome Trafficking to Mitotic Centrosome Upstream of *Fip5* Function

**Figure 7:**
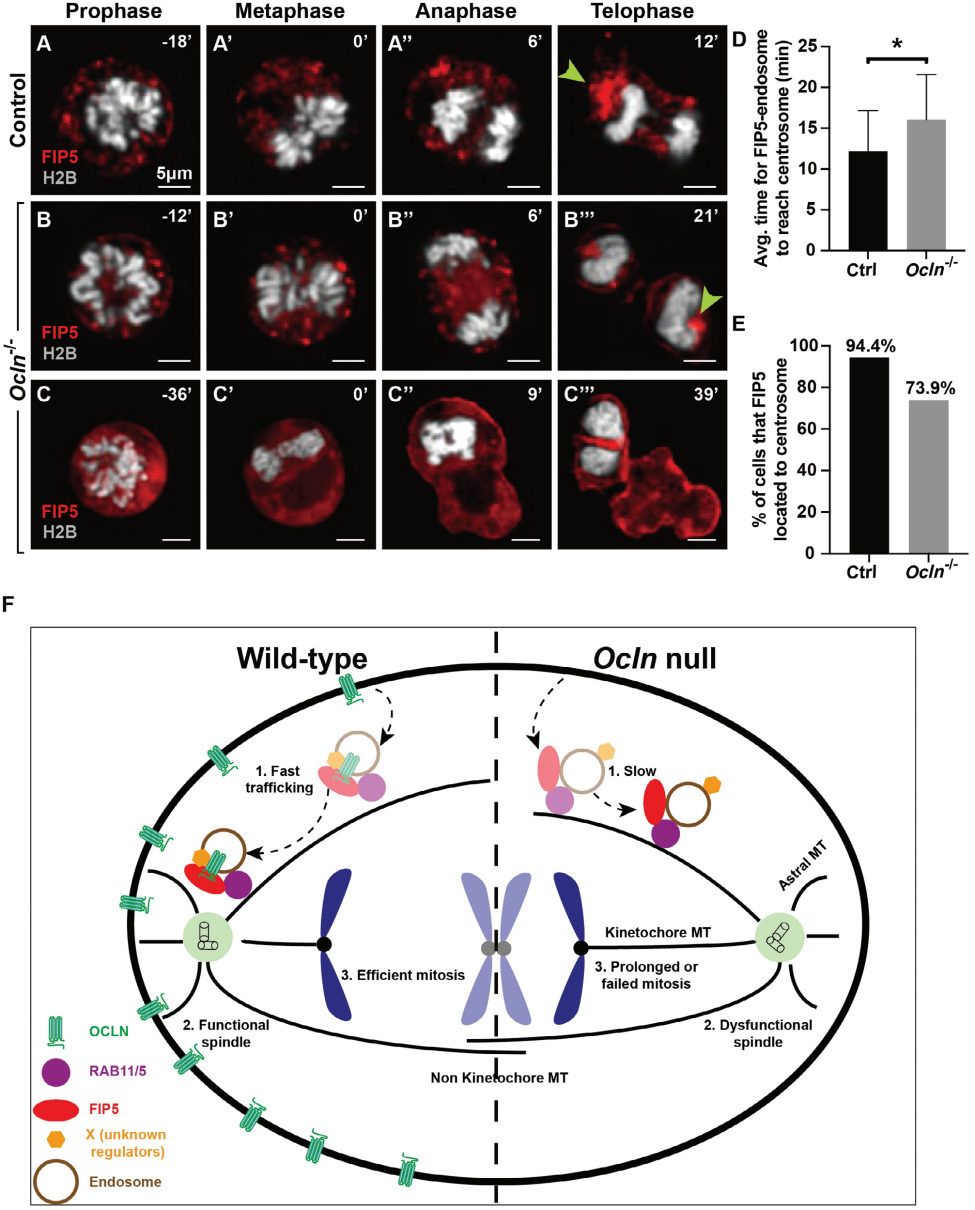
*Ocln* promotes endosome trafficking to the mitotic centrosome upstream of the *Fip5* function. A–C) Effects of *Ocln* loss on the trafficking of *Fip5*‐positive endosome to the centrosome as revealed by time‐lapse video recording in control (A‐A’’’) and *Ocln* null (B‐B’’’, C‐C’’’) HC11 cells. FIP5 was fused in‐frame with mCherry (red) at the N‐terminus. Arrowheads indicate mitotic endosomes surrounding the centrosomes. Note that in some *Ocln* null cells, *Fip5* protein became present on the cell surface (C). D,E) Quantification of the increase in fluorescent intensity at the centrosome (D) and percentages of cells in which FIP5‐positive particles/vesicles successfully congregated at the centrosome (E). Chromosomes were marked by H2B‐eGFP (white). Scale bars: 5 µm. **F**) Schematic diagram of a model depicting OCLN regulation of mitosis. OCLN and FIP5 facilitate the trafficking of mitotic endosomes toward the centrosome around the spindle pole, presumably via binding to the mitotic endosome regulator RAB11, with which FIP5 is known to bind. This process is essential for proper spindle assembly and function, including the formation of astral microtubules, and regulation of spindle angle, to ensure a smooth and efficient progression to metaphase, and subsequent nuclear and cytoplasmic division. In the absence of OCLN, trafficking of mitotic endosomes to the centrosome/pole is impaired, resulting in defective spindle assembly, including the formation of shortened astral microtubules, and tilted mitotic spindle. As a consequence of the formation of a dysfunctional mitotic spindle in *Ocln* null cells, prophase is delayed and, in some cases, fails to reach metaphase, leading to prolonged or failed mitosis, including incomplete nuclear/cytoplasmic division. Note, that the protein partners of OCLN, identified in the current study, are candidates of the unknown essential regulators (X proteins) of mitosis carried by the endosomes.

To determine the functional relationship between *Ocln* and *Fip5*, we sought to examine the consequence of their respective loss on each other and endosome trafficking. Thus, we transfected *Ocln* null HC11 cells with a lentivirus construct expressing mCherry‐*Fip5*. We found that FIP5 protein was expressed in *Ocln* null cells similar to control cells (Figure **7**A,B). However, in some of *Ocln* null cells (≈26.1%, see below), FIP5 failed to localize to its usual places, especially to the peri‐centrosomal locations, but instead was found on the cell surface (compare Figure 7C to A,B; Movies S10 and S11, Supporting Information). We then quantified the average time taken by FIP5‐positive endosomes to reach the centrosome. Interestingly, we found that, while in control cells it took endosomes ≈12 mins to reach the centrosomes, in *Ocln* null cells they took 17 mins, or a 41.6% increase (Figure 7A,B′″,D). Furthermore, while we observed mitotic endosomes trafficking to the centrosome in 94.4% of control cells, only 73.9% of *Ocln* null cells showed this event (Figure 7E). The data thus suggest that *Ocln* regulates FIP5‐positive endosome trafficking to the centrosome.

Likewise, we found that OCLN protein was expressed in *Fip5* knockdown cells similar to that in control cells, suggesting that *Fip5* does not regulate OCLN protein expression. However, we found that *Fip5* knockdown did not affect OCLN‐positive endosome trafficking to the centrosome (Figure S7A–C; Movie S12, Supporting Information).

Together, these data suggest that *Ocln* promotes endosome trafficking upstream of *Fip5* during mitosis in mammary gland luminal cells.

## 3 Discussion

In the current study, we demonstrate that mammary glands lacking *Ocln* exhibit retarded branching due to reduced cell proliferation. Interestingly, *Ocln* regulates spindle orientation and function, and its loss leads to a range of phenotypes, including prolonged prophase and failed nuclear and/or cytoplasmic division. Mechanistically, OCLN binds to FIP5 and recruits recycling endosomes to the centrosome to participate in spindle assembly and function. Our results unveil a novel role in OCLN‐mediated endosomal trafficking and potentially highlight its involvement in mediating membranous vesicle trafficking and function.

### 3.1 Occludin Binds to *Fip5* to Regulate Endosome Trafficking and Mitotic Spindle Function

Our data demonstrate that the binding of OCLN and FIP5 regulates spindle organization and function through the involvement of mitotic endosomes. Consequently, both OCLN and FIP5 facilitate the trafficking of mitotic endosomes toward the centrosome around the spindle pole, presumably via binding to RAB11, which regulates mitotic endosome assembly and function and with which FIP5 is known to bind.^[^ ^21^ ^]^ This process is essential for proper spindle assembly and function, including the formation of astral microtubules, and regulation of spindle angle, to ensure a smooth and efficient progression to metaphase, and subsequent nuclear and cytoplasmic divisions (Figure 7F). In the absence of OCLN, trafficking of mitotic endosomes to the centrosome/pole is impaired, resulting in defective spindle assembly, including the formation of shortened astral microtubules, and tilted mitotic spindle. As a consequence of the formation of a dysfunctional mitotic spindle in *Ocln* null cells, prophase is delayed and, in some cases, fails to reach metaphase, leading to prolonged or failed mitosis, including incomplete nuclear/cytoplasmic division (Figure 7F).

Our results showing OCLN regulation of endosome trafficking to the centrosome are consistent with and provide a mechanistic explanation for previous reports showing its centrosome localization.^[^ ^9^, ^26^ ^]^ Our data demonstrating OCLN promotion of cell proliferation in the luminal epithelium of the mammary gland also align with its role in the neocortex during embryonic brain development,^[^ ^9^ ^]^ suggesting a common function in different developmental contexts. However, neither NuMN, an essential regulator of mitotic spindle function that was proposed to interact with OCLN in a previous study,^[^ ^9^ ^]^ nor any components of the centrosome, kinetochore, and spindle machinery were identified in our BioID assay by which to find OCLN's binding partners.

Together, the data suggest that OCLN is unlikely to directly participate in binding to the core mitotic spindle machinery to regulate its assembly and function.

### 3.2 Molecular Basis of Endosomal Regulation of Mitotic Spindle Function

Mitotic RABs, including RAB5 and RAB11, did not appear in our BioID assay. However, we found that OCLN binds to and colocalizes with FIP5, a known adaptor of RAB11 in various developmental settings.^[^ ^24^ ^]^ Our data show that similar to OCLN, FIP5 also plays an essential role in promoting mitotic progression during cell proliferation and epithelial morphogenesis of the mammary gland in vivo. The data are thus consistent with the role of RAB11 endosome in mitosis and FIP5 interaction with RAB11. At present, however, it has remained unclear whether OCLN regulation of mitotic endosome trafficking depends solely on RAB11, which we show co‐localizes with OCLN, via mutual interactions with FIP5, or on RAB5 as well, which also regulates mitosis and co‐localizes with OCLN. The latter scenario could be likely, as we anticipate future studies to address, if FIP5 interacts with RAB5, as it does with some RABs other than RAB11 in certain situations.^[^ ^24^ ^]^

The discovery that RAB11‐mediated endosome trafficking is essential for mitotic spindle assembly and function came as a big surprise to the cell biology community around ten years ago.^[^ ^21^, ^27^ ^]^ Until then, it was generally accepted that RAB‐mediated endosome trafficking is essential for mitosis, but only for its late‐stage during cytokinesis, in which de novo membrane formation is required to separate two daughter cells.^[^ ^24^ ^]^ An exciting implication of the discovery is that cargo proteins of the mitotic endosomes, which are largely unknown, are likely essential regulators that participate in spindle assembly and function.

In addition to fortuitously discovering a novel mechanism by which mitotic endosome trafficking is regulated, thus providing an important update to the above earlier studies, our report here potentially heralds a new era of understanding the basis of mitotic endosomes. Specifically, we have uncovered various binding partners of OCLN from the BioID assay, which we predict to be essential regulators for mitotic endosome function. We await with much excitement to validate their functions in future studies.

### 3.3 *Ocln* Function in Vertebrate Emergence

Based on what we know about OCLN function so far, can we speculate on what it does that is essential for vertebrate emergence during evolution? OCLN's role in cell proliferation, which has now been conclusively demonstrated in the neocortex of the brain^[^ ^9^ ^]^ and the mammary gland as we report here, is unlikely to be a contributing factor to vertebrate emergence because cell proliferation is not a defining feature of vertebrate cells when compared with invertebrate cells. How about its role in regulating the secretion of protein and lipids as we recently showed?^[^ ^10^, ^11^ ^]^ Considering that vertebrate cells do differentiate from invertebrate cells by having much more diversified secretory cells and systems, including the excretory, endocrine, and, especially, immune systems, this could be a plausible scenario.

Alternatively, is there a more unifying, fundamental, subcellular function than proliferation or secretion that OCLN performs in these different cells, organs, and organ systems? If we can extract the most conserved and fundamental function that OCLN plays in different cellular contexts, then we are likely to have come closer to understanding its role in facilitating vertebrate emergence. In this regard, it is tantalizing to speculate that OCLN's role in regulating membrane dynamics, essential for vesicular trafficking, fusion, secretion, etc., maybe one of its most fundamental roles. We await with excitement future studies to address these interesting questions.

## 4 Experimental Section

### Mouse Strain

Mice carrying the *Ocln* allele were genotyped as previously described.^[^ ^7^ ^]^ Wild‐type C57BL/6JNifdc mice and BALB/c Nude mice were purchased from Zhejiang Vital River Laboratory Animal Technology Co., Ltd. The mice were housed and maintained in accordance with regulations from the University of South China and ShanghaiTech University's Institutional Animal Care and Use Committee (IACUC# 20200713003).

### Mammary Gland Transplantation, Harvest, and Visualization

For mammary gland regeneration transplantation, three‐week‐old female nude mice were used, and a total of 20 000 mammary gland epithelial cells were injected into cleared fat pad, mammary glands were collected after waiting 4–5 weeks. Mammary glands were harvested and mounted on glass slides. The slides were placed in freshly prepared Carnoy's solution (absolute ethanol: acetic acid = 3:1) and fixed overnight at 4 °C. Concentration gradients of alcohol (70%, 35%, 17%, 0%) were used for rehydration. Then, the mammary glands were stained with Carmine red, cleared in Histoclear^,[^ ^15^ ^]^ and hyalinized in xylol after dehydration. The slides were photographed using a Zeiss Axio Zoom V16 stereoscope.

### Preparation of Mammary Gland Epithelial Cells and Fluorescence‐Activated Cell Sorting (FACS)

Extraction of primary mammary gland organoids was performed as previously described.^[^ ^28^ ^]^ Briefly, mouse # 2, 3, 4, and 5 mammary glands were collected and cut into 1 mm^3^. They were then digested for 30 min at 37 °C in 10 mL collagenase solution (DMEM/F12 (Gibco, #C11330500CP), containing 2 mg mL^−1^ collagenase (Sigma, #C5138), 2 mg mL^−1^ Trypsin (Gibco, #27250018), 5 µg mL^−1^ insulin (Yeasen, #40107ES25), 5% fetal bovine serum (FBS) (Lonza, #S711‐001S)). Mammary organoids were pelleted by centrifugation at 450 g for 10 min and then purified by 3–5 rounds of differential centrifugations. Mammary gland organoids were digested in a 0.25% Trypsin‐EDTA (Meilunbio, #MA0233) water bath for 12 min into single cells for subsequent sorting or virus infection.

For FACS, Mammary gland epithelial single cells were centrifuged at 1000 g for 3 min and resuspended in FACS buffer (2% FBS in PBS) with antibodies against CD24‐eFluor450 (Invitrogen, #48‐0242‐82) and CD49f‐APC (Invitrogen, #17‐0495‐82). DAPI or Fixable Viability Dye eFluor 780 (Invitrogen, #65‐0865‐14) was used for sorting live cells. The luminal cells and basal cells were CD24^high^CD49f^low^ and CD24^low^CD49f^high^, respectively. FlowJo was used for analyzing the luminal to basal cell ratio.

### In Vitro Mammary Epithelial Branching and Migration Assays

For branching experiments, mammary gland organoids were mixed with 100% GFR (Growth Factor Reduced) Matrigel (Corning, #354230) and planted in a 24‐well plate preheated to 37 °C for 8 min. Then, add 1 mL of basic medium (DMEM/F12 with 1x ITS (Gibco, #41400‐045) and 1% P/S (Gibco, #15140‐122)) containing FGF2 (FGF2 concentration gradients: 0, 0.125, 0.25, 0.5, 1 nM). The organoids were cultured at 37 °C with 5% CO_2_ and imaged at 2, 39, 50, and 74 h. For breast organoid migration assays, refer to Star Protocol.^[^ ^29^ ^]^

### Luminal Cells Transcriptome Sequencing and Bioinformatics Analysis

Luminal cells were extracted from control and *Ocln* ^−/−^ mice at the 7‐week stage. Then, RNA was isolated using an RNA extraction kit (Magen, #R4012‐03). Total RNA was subjected to Bulk RNA‐seq by GENEWIZ. For bioinformatics analysis, differential genes were analyzed using the DESeq2 package.^[^ ^30^ ^]^ For GO enrichment analysis and GSEA, the clusterProfiler R package was utilized.^[^ ^31^ ^]^

### Cell Cycle Analysis

Luminal cells were extracted and cultured in 5% Matrigel at 37 °C with 5% CO_2_ for 3 days. Then, the luminal organoids were dissociated into single cells with 0.25% Trypsin‐EDTA. The single cells were washed once with PBS and fixed in pre‐cooled 70% ethanol for 2 h or overnight at 4 °C. The cell cycle and apoptosis analysis kit (YEASEN, #40301) was used to analyze the cell cycle, and FlowJo was used for cell cycle analysis.

### Cell Culture

HC11 cells were cultured in RPMI 1640 (Gibco, #C11875500CP), supplemented with 10% FBS, 5 µg mL^−1^ insulin, 10 ng mL^−1^ EGF (GenScript, #Z02691), 1% L‐Ala‐Gln (Beyotime, #C0211), and 1% P/S. HEK293T cells were cultured in DMEM (Gibco, #C12430500BT), supplemented with 10% FBS, 1% sodium pyruvate (Beyotime, #C0331), 1% L‐Ala‐Gln, 1% non‐essential amino acids (Beyotime, #C0332), and 1% P/S. Primary luminal cells were cultured in advanced DMEM/F12 (Gibco, #12 634 010) supplemented with 5% FBS (Gibco, #10099141), 10 ng mL^−1^ EGF, 20 ng mL^−1^ FGF2 (GenScript, #Z03116), 5 µM Y27632 (Sellect, S1049), 4 µg mL^−1^ Heparin (Thermo, #A16198), 1% sodium pyruvate, 1% L‐Ala‐Gln, 1% P/S, and 5% Matrigel (Corning, #354234). All cells were confirmed to be free of mycoplasma by PCR.

### Virus Construction and Packaging

Sufficient HEK293T cells and plasmids, including psPax2 (Addgene #12260) and pMD2 (Addgene #12259), as well as transfer plasmids, were prepared. Then, EZtrans (Shanghai Life‐iLab Biotech Co., Ltd, #AC04L099) was used for plasmid transfection for 12–16 h. The medium should be replaced with a fresh medium after transfection. Lentivirus‐containing medium was harvested at 48 and 72 h, and then ultracentrifuged at 27 000 rpm for 2 h after filtering out cell debris. The lentivirus was suspended in a small amount of DMEM/F12 containing 10% FBS and stored at −80 °C.

### Proximity‐Dependent Biotin Identification (BioID)

Luminal cells were infected with BirA empty and BirA‐*Ocln* lentivirus, respectively. The cells were cultured in Matrigel for two generations to obtain enough infected cells. Subsequently, the medium was changed into a fresh one containing 50 µM Biotin (BBI, #A600078‐0001). After 24 h, the cells were harvested, lysed with 1% NP40, and biotin‐labeled proteins were enriched using NeutrAvidin Agarose (Thermo, #29200) for mass spectrometry (MS).

For MS, western blot and Coomassie Blue staining are performed. The gel was cut into pieces with 3mm^3^ size, which was alternately bathed in 25 mM NH_4_HCO_3_/acetonitrile (ACN) (1:1) and 25 mM NH_4_HCO_3_ until decoloration. Subsequently, dithiothreitol and iodoacetamide were used to fully denature the protein. The gel particles were then digested with 0.5 µg µL^−1^ protease at 37 °C for 12–16 h. Formic acid was used to elute short peptides. An Orbitrap Fusion Mass Spectrometer was used for analysis.

### Co‐Immunoprecipitation (Co‐IP) Assay and Western Bolt Assay

For the Co‐IP assay, HEK293T cells expressing FLAG‐OCLN and HA‐FIP5 were harvested and washed twice with PBS. The cells were then lysed with 1% NP40 buffer. The lysate was co‐incubated with FLAG‐tagged magnetic beads (Bimake, #B26101) at 4 °C overnight. The beads were washed three times with TBST. After that, the FLAG‐tagged proteins were eluted with 40 µL loading buffer for subsequent western blot analysis.

In the western blot assay, standard protocols were followed. The antibodies were as follows: anti‐FLAG (Cell Signaling Technology, #2368), and anti‐HA (Cell Signaling Technology, #2999). Imaging was conducted using an Amersham Imager 680.

### Immunofluorescence and Confocal Microscopy

For immunofluorescence analysis, HC11 cells transfected with eGFP‐OCLN lentivirus were plated into an 8‐well chamber for 16 h. The cells were blocked for 1 h at room temperature (RT) in PBS containing 10% goat serum and 0.2% Tween 20 and then were incubated with a primary antibody at 4 °C overnight. Subsequently, a secondary antibody was applied at RT for 2 h, and DAPI was used for nuclear staining. The mitotic spindle angle was determined by measuring the angle between centrosomes (marked by γ‐tubulin immunofluorescence in red) in the horizontal X‐Y plane and vertical X‐Z plane. The antibodies used in this study were as follow: anti‐Rab11 antibody (Cell Signaling, #5589T, 1:200 dilution), anti‐Rab7 antibody (Cell Signaling, #9367T, 1:200 dilution), anti‐Rab5 antibody (Cell Signaling, #3547T, 1:200 dilution), anti‐γ‐tubulin antibody (Sigma, #T5192, 1:200 dilution), anti‐transferrin receptor (TfR, anti‐mouse CD71) antibody (BioLegend, #113802, 1:50 dilution), anti‐α‐tubulin antibody (Sigma, #T9026, 1:200 dilution). Confocal imaging was performed using a ZEISS LSM980 Airyscan confocal microscope.

### Quantitative Real‐Time PCR

Trizol reagent (Vazyme, #R401‐01) was used to extract total RNA from basal and luminal cells in the mammary gland. Then, the cDNA was prepared with equal amounts of RNA templates, and the qPCR was performed using SYBR green reactions on the BIO‐RAD CFX Connect Real‐Time System, following the manufacturer's protocol. Relative expression levels were calculated using the comparative CT method. Data were normalized to the expression of *Actb*. The primers were as follows:

*Actb*: Forward 5′‐ggctgtattcccctccatcg‐3′, Reverse 5′‐ccagttggtaacaatgccatgt‐3′;

*Etv4*: Forward 5′‐cggaggatgaaaggcggatac‐3′, Reverse 5′‐tcttggaagtgactgaggtcc‐3′;

*Evt5*: Forward 5′‐tcagtctgataacttggtgcttc‐3′, Reverse 5′‐ggcttcctatcgtaggcacaa‐3′;

*Mkp3*: Forward 5′‐tcgggctgctgctcaagaaac‐3′, Reverse 5′‐cggtcaaggtcagactcaatgtcc‐3′;

*Rab11fip5*: Forward 5′‐ctctggacgaggtcttccg‐3′, Reverse 5′‐tgttccgtgtgaactggatgg‐3′.

### shRNA‐Mediated Knockdown of Gene Expression

The design of shRNAs was based on an online tool at Thermo Fisher (https://rnaidesigner.thermofisher.com/rnaiexpress/sort.do). shRNA fragments were then cloned into a modified version of the pLKO.1‐GFP vector (Addgene #8453). HEK293T cells were used for lentiviral production, following a standard protocol. The lentivirus was concentrated and was used to infect HC11 cells and luminal cells. Infected cells were selected using FACS, and the positive cells were subsequently cultured for live imaging and cell cycle detection. The shRNA sequences were as follows:

Scramble shRNA (shScrb): 5′‐acctaaggttaagtcgccctcg‐3′.

*Rab11Fip5* shRNA (sh*Fip5*): 5′‐gagcgccagcatgtttgatct‐3′.

### CRISPR‐Mediated Gene Functional Knockouts and Tagging

For CRISPR knockout, a CRISPR lentivirus containing *Ocln* sgRNA was transfected into HC11 cells. Subsequently, monoclonal cells were sorted into 96‐well plates. Western blotting and sanger sequencing are used to screen the positive monoclones.

For CRISPR‐mediated tagging, two constructs containing CRISPR/Cas9 with *H2b* sgRNA and its homologous region with eGFP sequences were transfected into HC11 cells. The cells were plated into a 10‐cm dish for 3 days, and then eGFP‐positive cells were isolated by FACS. Importantly, the H2B‐eGFP‐*Ocln* ^−/−^ HC11 cell line was established on the basis of the H2B‐eGFP HC11 cell line. The sgRNAs were designed on the E‐CRISP website (http://www.e‐crisp.org/E‐CRISP/designcrispr.html).

*Ocln* sgRNA: 5′‐gcaccaagcaatgacatgta‐3′. *H2b* sgRNA: 5′‐gcgtttgaccacagccaggt‐3′.

### Live Cell Imaging and Co‐Localization Assay

For the co‐localization assay, H2B‐eGFP HC11 cells overexpressing both BFP‐OCLN and mCherry‐FIP5 were seeded into an 8‐well chamber for 16 h and then visualized using a Nikon TI2‐E microscope with CSU W1 Sora 1 Camera for time‐lapse imaging. For other live cell imaging assays, BFP‐OCLN, mCherry‐OCLN, mCherry‐FIP5, shScrb, and sh*Fip5* lentivirus were utilized. Tubulin was labeled with the SPY650‐tubulin probe (Cytoskeleton, #CY‐SC503). ImageJ Fiji was employed for analysis.

### Statistics

The sample sizes for the data in each figure are indicated in the figure legends. Statistical significance between conditions was assessed using two‐tailed Student's *t*‐tests. All error bars represent the standard deviation (SD), and significance is denoted ^*^ *p* < 0.05, ^**^ *p* < 0.01, ^***^ *p* < 0.001, and ^****^ *p* < 0.0001. “ns” denotes not significant.

## Author Contributions

Z.Z.performed almost all the experiments, except those stated below. J.C. performed assistance with both fluorescent compound microscopy, live cell imaging, and data analysis. R.M. performed quality control, diagrams, and review. C.X. performed wholemount imaging of Carmine‐stained mammary glands showing retarded branching phenotype. Y.L. performed data interpretation and experimental design. J.C., Z.Z., and K.X. performed intellectual contributions and reviews. P.L. performed conceptualization, funding acquisition, data curation, and writing.

## Conflict of Interest

The authors declare no conflict of interest.

## Supporting information

Supporting Information

Supplemental Movie 1

Supplemental Movie 2

Supplemental Movie 3

Supplemental Movie 4

Supplemental Movie 5

Supplemental Movie 6

Supplemental Movie 7

Supplemental Movie 8

Supplemental Movie 9

Supplemental Movie 10

Supplemental Movie 11

Supplemental Movie 12

Supporting Information

## Data Availability

The data that support the findings of this study are available from the corresponding author upon reasonable request.
