# Supplementary material for: Tight Junction Component Occludin Binds to FIP5 to Regulate Endosome Trafficking and Mitotic Spindle Function
Source: Adv Sci (Weinh). 2024 Jun 17;11(30):2308822. doi: 10.1002/advs.202308822 (PMC11321699; doi:10.1002/advs.202308822)

ADVANCED SCIENCE
Open Access

Supporting Information

for *Adv. Sci.*, DOI 10.1002/advs.202308822

Tight Junction Component Occludin Binds to FIP5 to Regulate Endosome Trafficking and Mitotic Spindle Function

*Zichao Zhang, Jing Chen, Rongze Ma, Chongshen Xu, Yunzhe Lu, Jiecan Zhou, Kun Xia and Pengfei Lu**

SUPPLEMENTARY FIGURE LEGENDS

Supplementary Figure 1: *Ocln* promotes epithelial expansion during mammary gland branching morphogenesis.

(**A**-**C**) Relative mRNA expression of the FGF signaling target genes *Etv4* (**A**), *Etv5* (**B**), and *Mkp3* (**C**) in control and *Ocln* null primary luminal cells. The expression is relative to that of the control samples. Data are presented as mean ± SD. Statistical analysis was performed using an unpaired Student t-test. "ns" indicates no significance.

Supplementary Figure 2: *Ocln* regulates mitosis of luminal epithelial cells.

(**A**, **B**) Gating strategy for sorting mammary gland cell populations based on CD24 and Itga6 (CD49f) by FACS. $CD24^{hi}CD49f^{low}$ represented luminal cells (Lu), $CD24^{med}CD49f^{hi}$ represented basal cells (Ba).

(**C**) Cell number ratio of luminal cells to basal cells. (control, n = 9; *Ocln*$^{-/-}$, n = 8). A *t*-test was used; *P < 0.05.

Supplementary Figure 3: CRIPR-based *Ocln* knockout and validation in HC11 cells.

(**A**) Western blotting showing the absence of OCLN protein expression in three clones of HC11 cells. (**B**) Sequencing showing the point mutation by insertion in the KO1 clone of HC11 cells.

Supplementary Figure 4: OCLN is a resident component in the mitotic endosomes.

(**A-I'''**) Co-localization analysis of TfR (red, **A-A'''**), RAB5 (red, **D-D'''**), RAB7 (red, **G-G'''**), and OCLN (eGFP-OCLN, green, **B-B'''**, **E-E'''**, and **H-H'''**) using a time course of fluorescent confocal imaging during mitosis. Note that chromosomes were marked by DAPI fluorescence (white) in the overlaid images (**C-C'''**, **F-F'''**, and **I-I'''**). Scale bars: 5 µm.

Supplementary Figure 5: OCLN binds to the vesicular trafficking regulator FIP5.

(**A**) Gene Ontology analysis of the differentially (P<0.05) expressed genes (detailed list in Supplementary Table 1) was conducted to determine the biological processes associated with these genes. (**B**, **C**) Categorization of OCLN-binding partners based on their supposed functions (**B**). (**C**) Detailed list of OCLN-binding partners in the Protein transport and trafficking category, which includes the RAB11-binding protein FIP5.

Supplementary Figure 6: *Fip5* regulates mitotic spindle function and epithelial branching in

the mammary gland.

(**A**-**B**) Quantification of spindle angles (**A**) or angle distributions (B) in control (shScrb) and *Fip5* (sh*Fip5*) knockdown cells. (shScrb, n = 36; sh*Fip5*, n = 45). A *t*-test was used. n.s. (not significant).

(**C**-**E**) Cell cycle analysis of control (**C**) and *Fip5* knockdown (**D**) luminal epithelial cells based on flow cytometry. (**E**) Quantification of the percentages of cell numbers in different phases of the cell cycle (both shScrb and sh*Fip5*, n = 6). A *t*-test was used. *, P<0.05; **, P<0.01; ***, P<0.001.

(**F**, **G**) *Fip5* mRNA expression as detected by qPCR (**F**) and cell cycle analysis (**G**) of control cells, *shFip5* cells, *shFip5* cells overexpressing *Fip5*. A *t*-test was used. **, P<0.01; ***, P<0.001.

Supplementary Figure 7: *Ocln* promotes endosome trafficking to mitotic centrosome upstream of *Fip5* function.

(**A**-**C**) Effects of *Fip5* knockdown on the trafficking of OCLN-positive endosome to the centrosome as revealed by time-lapse video recording in control (**A**-**A'''**) and *Fip5* knockdown (**B**-**B'''**) HC11 cells. Green arrowheads indicate mitotic endosomes surrounding the centrosome. (**C**) Quantification of the increase in fluorescent intensity at the centrosome. Chromosomes were marked by H2B-eGFP (white). A *t*-test was used. n.s., not significant. Scale bars: 5 μm.

Figure S1. *Ocln* promotes epithelial expansion during mammary gland branching morphogenesis.

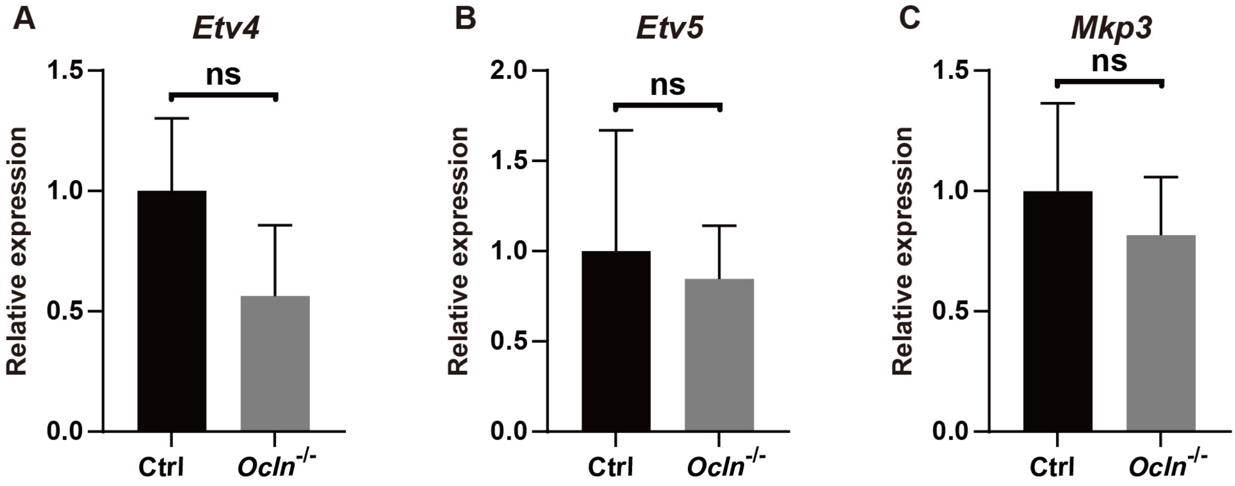

Figure S2. *Ocln* regulates mitosis of luminal epithelial cells.

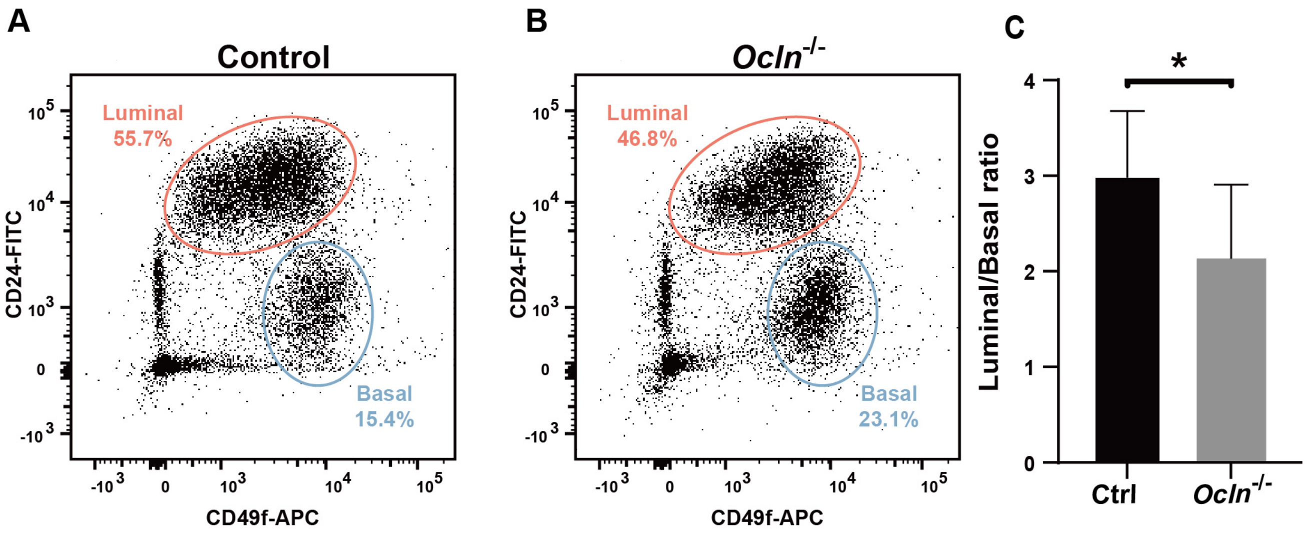

Figure S3. CRISPR-based *Ocln* knockout and validation.

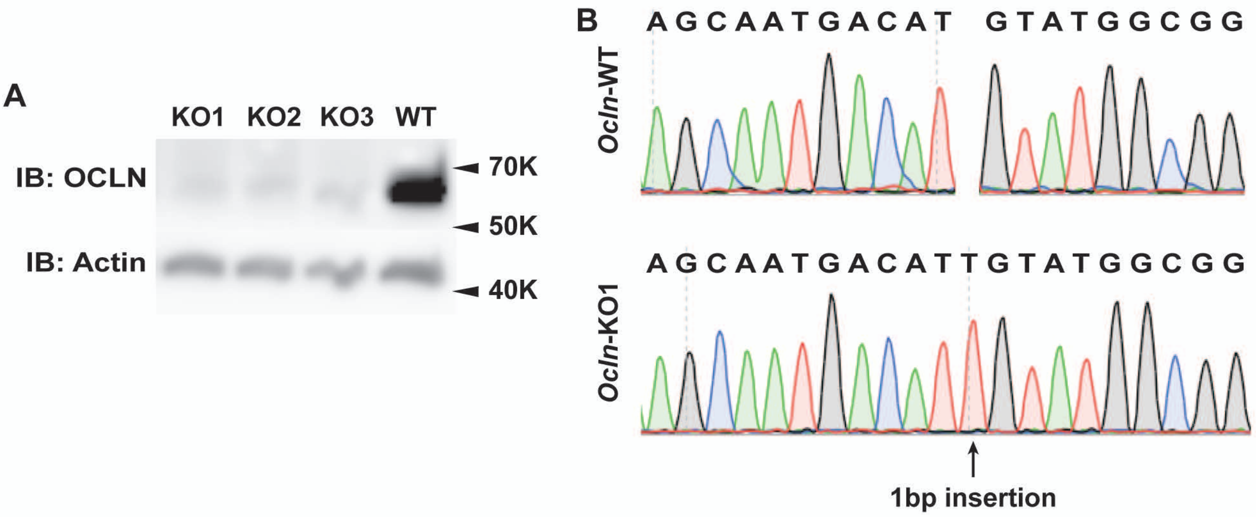

Figure S4. OCLN is a component of mitotic endosomes.

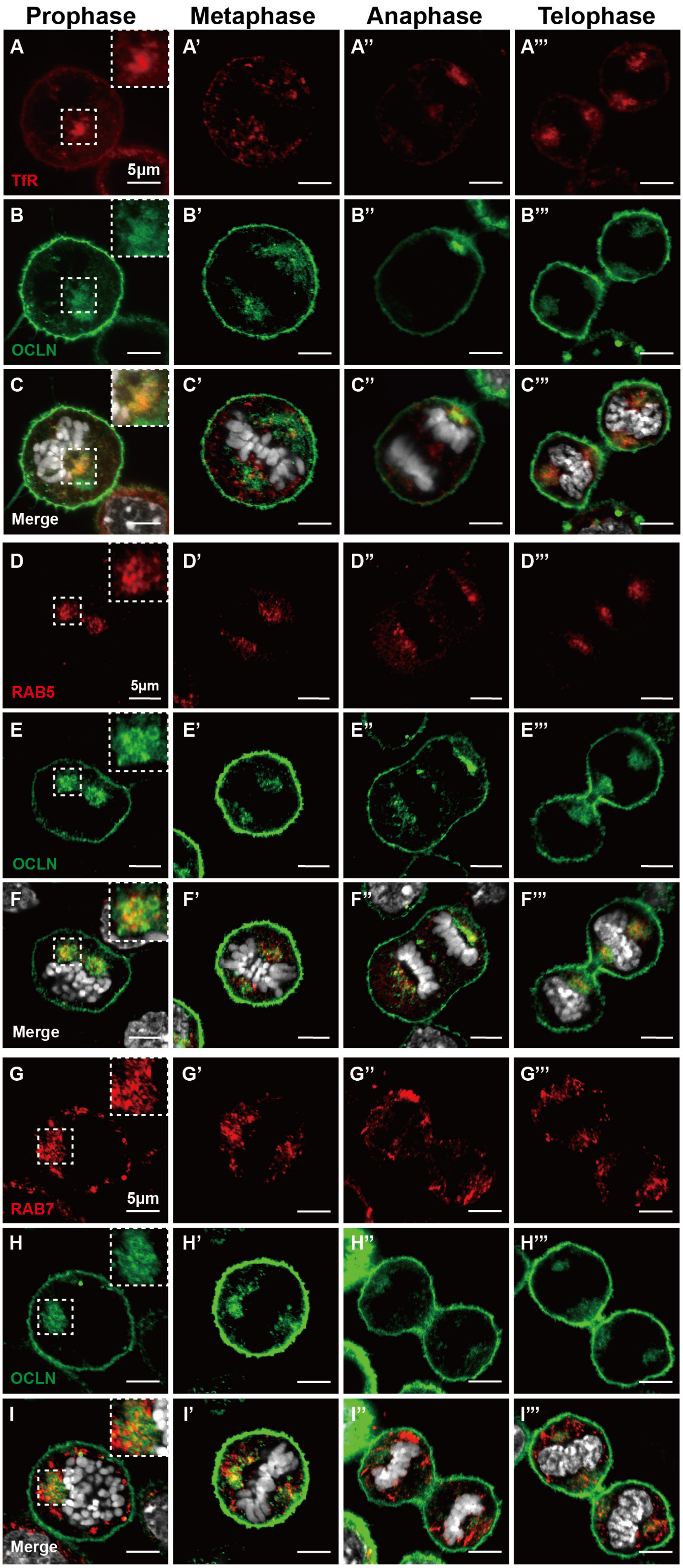

Figure S5. OCLN binds to the endosome trafficking regulator FIP5.

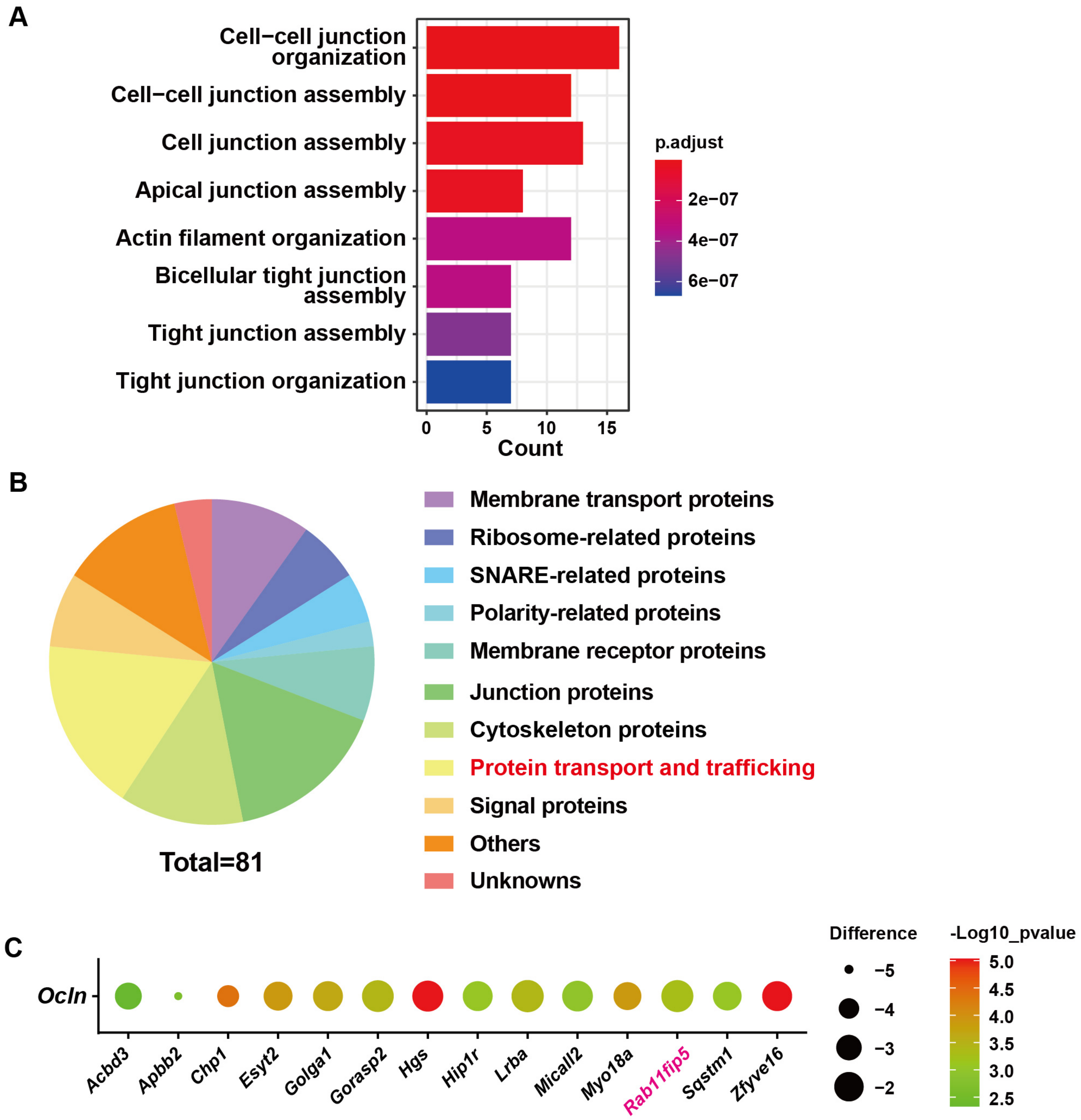

Figure S6. ***Fip5*** **regulates mitotic spindle function and epithelial branching in the mammary gland.**

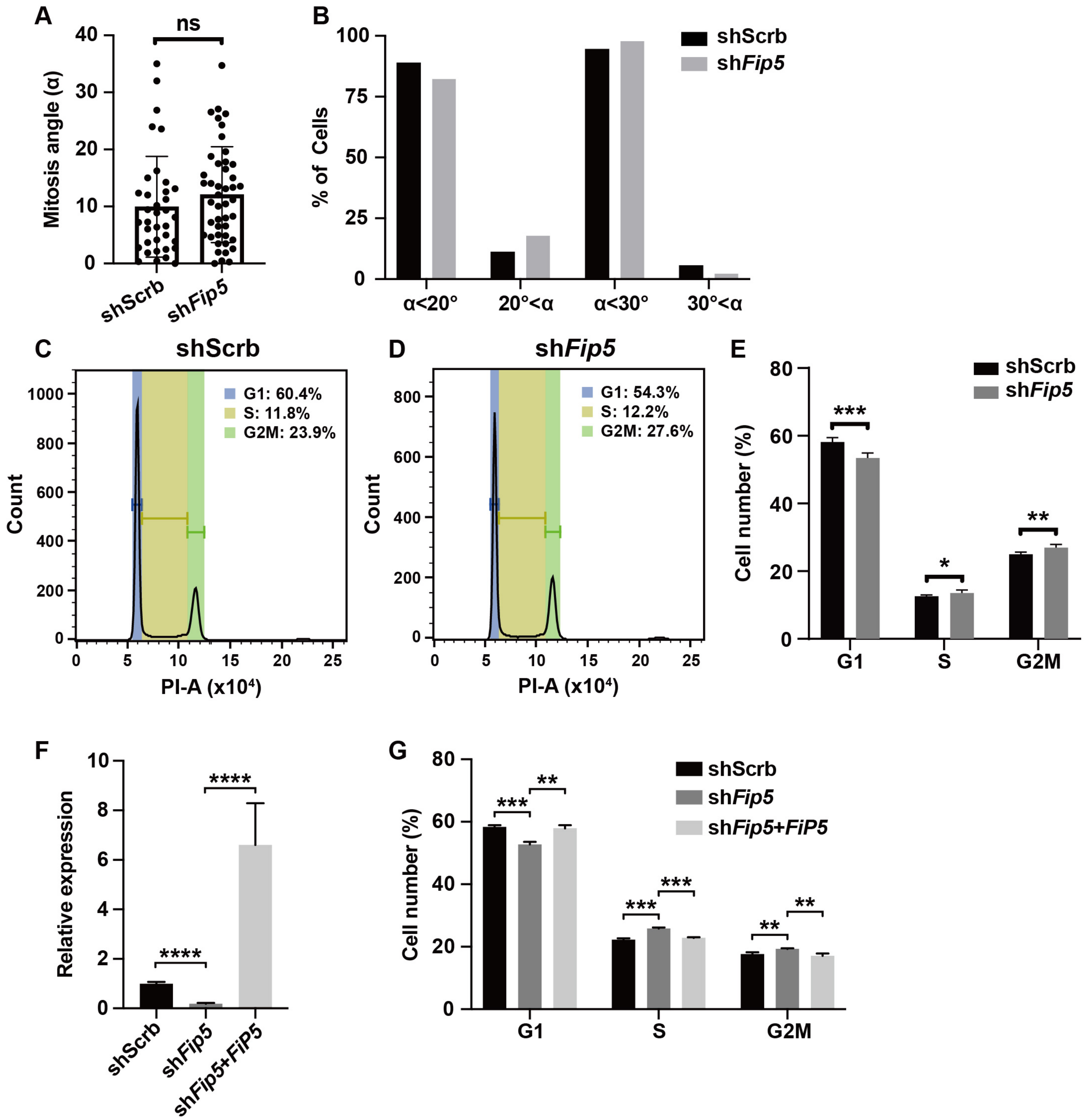

Figure S7. *Ocln* promotes endosome trafficking to mitotic centrosome upstream of *Fip5* function.

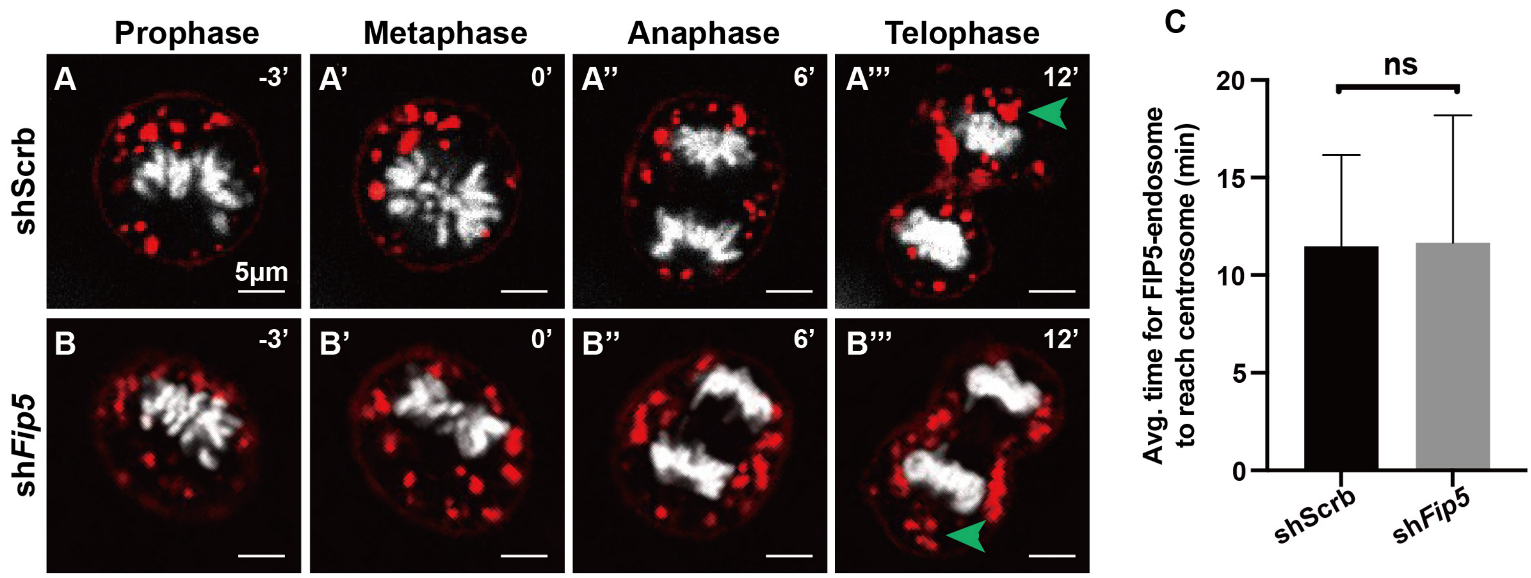

Supplement: Supplementary file 1 — Supporting Information [file ADVS-11-2308822-s008.pdf]
